# Molecular Identification and Survey of *Cyclospora* spp. in Cattle in Shanxi Province, North China

**DOI:** 10.3390/ani14142114

**Published:** 2024-07-20

**Authors:** Ze-Xuan Wu, Yu Kang, Shi-Bo Huang, Ya-Ya Liu, Jin-Jin Mei, Qing Liu, Xing-Quan Zhu

**Affiliations:** Laboratory of Parasitic Diseases, College of Veterinary Medicine, Shanxi Agricultural University, Taigu, Jinzhong 030801, China; wuzexuan0602@163.com (Z.-X.W.); yukang2024@126.com (Y.K.); h17516211643@126.com (S.-B.H.); ly1397772866@163.com (Y.-Y.L.); mjj3051@163.com (J.-J.M.)

**Keywords:** cyclosporiasis, beef cattle, dairy cattle, prevalence, sequence analysis, Shanxi Province

## Abstract

**Simple Summary:**

*Cyclospora cayetanensis* is an emerging zoonotic intestinal protozoan that poses a threat to human health, and the main symptoms of *C. cayetanensis* infection are voluminous, watery diarrhea, abdominal cramps, nausea, low-grade fever, fatigue, and weight loss. However, to date, no study of *Cyclospora* spp. has been reported in cattle in Shanxi Province. We first reported the occurrence and prevalence of *Cyclospora* spp. in beef and dairy cattle in Shanxi Province, China. A total of 761 fecal samples collected from cattle in three representative counties in this Province were examined for *Cyclospora* spp. by using a polymerase-chain-reaction–restriction-fragment-length polymorphism (PCR–RFLP) test targeting the nuclear small subunit ribosomal RNA (SSU rRNA) gene. The prevalence of *Cyclospora* spp. in cattle was 2.1%, and region, age, sex, and breed were not found to be significantly associated with *Cyclospora* prevalence. Twelve representative sequences were obtained. Among them, seven sequences were identified as *Cyclospora colobi* and five sequences were identified as other *Cyclospora* spp.. This study reported the occurrence and prevalence of *Cyclospora* spp. in cattle in Shanxi Province for the first time, which extends the geographical distribution of the genus *Cyclospora*.

**Abstract:**

To date, more than 20 species in the genus *Cyclospora* have been reported. Among them, *Cyclospora cayetanensis* has been recognized as the causative agent of human cyclosporiasis, which is characterized by severe intestinal injury and prolonged diarrhea in patients with immune dysfunction. The presence of *C. cayetanensis* in cattle has been confirmed. To date, however, no surveillance data are available on the occurrence and prevalence of *Cyclospora* spp. in cattle in Shanxi Province, North China. In the present study, a total of 761 fecal samples collected from cattle in three representative counties (Qi, Jishan, and Shanyin) in this Province were examined for *Cyclospora* spp. by using a polymerase-chain-reaction–restriction-fragment-length polymorphism (PCR–RFLP) test based on the nuclear small subunit ribosomal RNA (SSU rRNA) gene. The prevalence of *Cyclospora* spp. in cattle was 2.1%, and region, age, sex, and breed were not identified to be risk factors. Molecular evolutionary analysis based on the SSU rRNA sequences revealed that all 12 of the isolates were relatively distant from the human pathogen *C. cayetanensis*; seven isolates were grouped with *Cyclospora colobi*, whereas the others were grouped with cattle *Cyclospora* spp. reported previously. Though *C. cayetanensis* was not detected in cattle in the present study, more investigations should be performed in human populations, other animal species, or cattle from other regions of Shanxi Province and other environmental sources from the One Health perspective.

## 1. Introduction

*Cyclospora* spp. are protozoan parasites classified within the phylum Apicomplexa [1]. The first published report on the existence of *Cyclospora* infection in humans can probably be dated to the end of the 1970s, though the authors concluded that the parasite detected in three patients was possibly a coccidian of the genus *Isospora* [2,3]. Afterwards, the organism has been described as coccidian-like body (CLB), cyanobacterium-like body, large *Cryptosporidium,* or blue-green algae [2,4]. Eventually, this new organism was classified into the genus *Cyclospora* and received its current name (*Cyclospora cayetanensis*) in the early 1990s [5,6]. *C. cayetanensis* has been recognized as the causative agent of an illness in humans called cyclosporiasis [1].

Thus far, more than 50 countries worldwide have documented the occurrence of *C. cayetanensis* infection in humans, and its prevalence was estimated to be 3.4% [7]. Notably, cyclosporiasis outbreaks have been reported in 13 countries [8]. The symptoms of cyclosporiasis vary depending on the age and immune status of the host [9]. Asymptomatic infections are common in residents of the endemic areas [2]. When present, clinical manifestations of cyclosporiasis include profuse, watery diarrhea, abdominal cramps, low-grade fever, nausea, anorexia, weight loss, and fatigue [9]. In healthy individuals, *C. cayetanensis* usually causes mild-to-moderate, self-limiting diarrhea [10]. However, severe intestinal injury and prolonged diarrhea have been reported in patients with immune dysfunction [8,10]. In addition, extraintestinal complications have also been reported, such as acalculous cholecystitis and ocular inflammation [11].

In animals, many species of the genus *Cyclospora* have been identified, such as *Cyclospora macacae*, *Cyclospora cercopitheci*, *Cyclospora papionis, Cyclospora colobi*, and *C. cayetanensis* [1,12,13]. Moreover, the presence of *Cyclospora* spp., including *C. cayetanensis*, has been documented in the feces of several animal species, including cattle [13,14,15,16]. Notably, contact with animals was identified as a risk factor for human infection with *Cyclospora* spp. [1,17]. Therefore, prevalence data on *Cyclospora* spp. in cattle are of great importance. Due to the rich natural grassland and crop straw resources in Shanxi Province, the development of high-quality beef cattle and dairy cows is becoming an important sector of its agriculture. To date, however, no data are available on the occurrence and prevalence of *Cyclospora* spp. in cattle in Shanxi Province, North China. Therefore, the present study aimed to determine the occurrence and prevalence of *Cyclospora* spp. in cattle in Shanxi Province by using the nested polymerase-chain-reaction–restriction-fragment-length polymorphism assay, followed by sequencing and phylogenetic analysis.

## 2. Materials and Methods

### 2.1. Study Sites and Sample Collection

Shanxi Province (34 36′–40 44′ N, 110 15′–114 32′ E), located in the east wing of the Loess Plateau in western North China, has a temperate continental monsoon climate with four distinct seasons. In November 2020, a total of 761 fecal samples were collected from dairy cattle and beef cattle in three counties in this province, namely Jishan, Qi, and Shanyin [18]. These three counties are located in southern, central, and northern Shanxi Province, respectively. Each sample (approximately 10 g) was collected from the top of freshly defecated, uncontaminated feces using polyethylene (PE) gloves, and information on the region, sex, and age was recorded for each animal. All fecal samples were transported to the Laboratory of Parasitic Diseases at Shanxi Agricultural University by storing them along with ice packs. Upon arrival, the fecal samples were preserved at −20 °C until DNA extraction.

### 2.2. DNA Extraction and PCR Amplification

An E.Z.N.A Stool DNA extraction kit (Omega Bio-tek Inc., Norcross, GA, USA) was used to extract the total genomic DNA from 0.2 g of each of the fecal samples described above, in accordance with the manufacturer’s specifications. Then, the DNA samples were kept frozen at −20 °C until they were used in the PCR analysis.

The DNA preparations were screened for the presence of *Cyclospora* spp. by nested PCR amplification of a fragment (approximately 500 bp) of the small subunit ribosomal RNA (SSU rRNA) gene, as previously described [19], with minor modifications. The first-round PCR was carried out using the primer set (forward: 5′-AATGTAAAACCCTTCCAGAGTAAC-3′; reverse: 5′-GCAATAATCTATCCCCATCACG-3′) with the following cycling conditions: an initial hot start at 94 °C for 7 min, followed by 35 cycles of 95 °C for 45 s, 55 °C for 45 s, 72 °C for 90 s, and a final extension at 72 °C for 10 min. The second-round PCR was performed using an internal primer set (forward: 5′-AATTCCAGCTCCAATAGTGTAT-3′; reverse: 5′-CAGGAGAAGCCAAGGTAGGCRTTT-3′), and the thermal cycling conditions were identical to the first-round PCR, except for the extension time (1 min).

PCR amplification was performed in a total reaction volume of 25 μL containing 2.5 μL 10 × PCR buffer (Mg^2+^ free), 200 μM of dNTP mixture, 2.0 mM MgCl_2_, 1 unit of TaKaRa Ex-*Taq* DNA polymerase, 0.4 μM of each primer, and 2 μL of genomic DNA for the primary PCR or the product obtained in the first-round PCR for the second-round PCR. The final PCR products were separated by 2.0% agarose gel electrophoresis for 30 min at a constant voltage of 120 V and visualized under ultraviolet (UV) light.

### 2.3. Restriction Fragment Length Polymorphism (RFLP) Assay

To differentiate *Cyclospora* spp. from *Eimeria* spp., an RFLP analysis was performed [14,15]. Briefly, according to the product manual, using restriction enzyme *Kpn*2I (*Bsp*EI) (NEB, Ipswich, MA, USA), the RFLP reaction mixture (with a final volume of 20 μL) consisted of 2.5 μL 10 × reaction buffer, 1 unit of restriction enzyme *Kpn*2I, and 12.5 µL of PCR products. Enzyme digestion was carried out at 55 °C for 12 h. Then, the digested solution was subjected to electrophoresis in a 2.0% agarose gel, followed by visualization under UV light.

### 2.4. Sequencing and Phylogenetic Analysis

Only PCR products successfully digested with *Kpn*2I were characterized by bi-directional Sanger sequencing at Sangon Biotech (Shanghai, China) using an Applied Biosystems™ 3730XL DNA Analyzer (Thermo Fisher Scientific, Waltham, MA, USA). Nucleotide sequences were edited and compared for similarity with those of the reference strains available in the GenBank database using the Basic Local Alignment Search Tool (BLAST) server. As described previously, a neighbor-joining (NJ) phylogenetic tree was constructed using MEGA 7.0 [15]. The Kimura parameter-2 model was used to compute the genetic distances between the sequences, and the robustness of the findings was evaluated by a bootstrap test with 1000 replicates [20].

### 2.5. Statistical Analysis

In the present study, a chi-squared (χ2) test was used to assess the association between the molecular prevalence of *Cyclospora* spp. and the risk factors using the software SPSS 26.0 (IBM, Chicago, IL, USA). The strength of association was evaluated by calculating odds ratios (ORs) with corresponding 95% confidence intervals (CIs). A *p*-value < 0.05 was considered indicative of a statistically significant difference.

## 3. Results

### 3.1. PCR Amplification and RFLP Analysis

**Figure 1:**
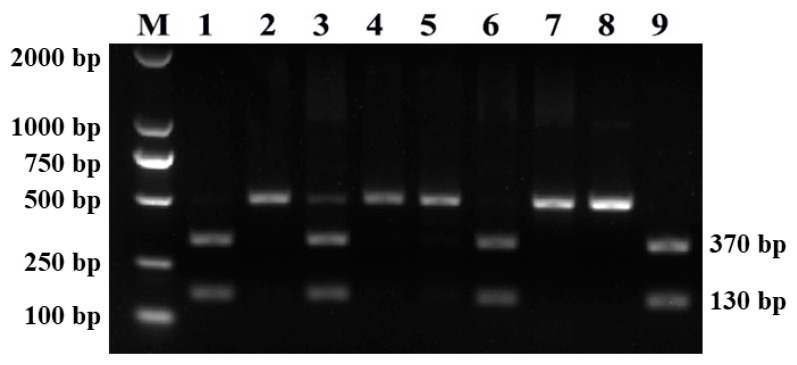
Restriction fragment length polymorphism (RFLP) analysis of the PCR products using *Kpn*2I (*Bsp*EI). Amplicons from *Eimeria* spp. were not digested (approximately 500 bp in length, lanes 2, 4, 5, 7, and 8), whereas the PCR amplicons amplified from the *Cyclospora* spp. were digested into two fragments (approximately 130 and 370 bp in length, respectively) (lanes 1, 6, and 9). One PCR amplicon showed three fragments after digestion (130, 370, and 500 bp, respectively) (lane 3), suggesting the co-infection of *Eimeria* spp. and *Cyclospora* spp..

The nested PCR results showed the presence of a specific band at the size of approximately 500 bp in 473 cattle fecal samples. Subsequently, the amplified PCR products were subjected to digestion with the restriction endonuclease *Kpn*2I, and the representative image is shown in Figure 1. The patterns were composed of one band of approximately 500 bp in length, two bands (approximately 130 bp and 370 bp in length, respectively), or three bands of different sizes. The digested fragments of the expected sizes (two fragments of 130 bp and 370 bp length, respectively) were obtained from 16 samples, and the overall prevalence of *Cyclospora* spp. was 2.1% (16/761) (Table 1).

### 3.2. Risk Factors of Cyclospora Infection

The analysis results of the possible risk factors of *Cyclospora* spp. infection in cattle in Shanxi Province are shown in Table 1. The highest prevalence of *Cyclospora* spp. in cattle was observed in Jishan County (3.3%, 9/273), followed by Qi County (1.7%, 5/288), and Shanyin County (1.0%, 2/200), but these differences were not statistically significant (*p* = 0.196). Also, there were no statistically significant differences in *Cyclospora* spp. prevalence in cattle between the two age groups (*p* = 0.053), nor between the two sex groups (*p* = 0.962). There were no statistically significant differences in the prevalence of *Cyclospora* spp. between the dairy cattle and the beef cattle (*p* = 0.580).

### 3.3. Sequence Alignment and Phylogenetic Analysis

**Figure 2:**
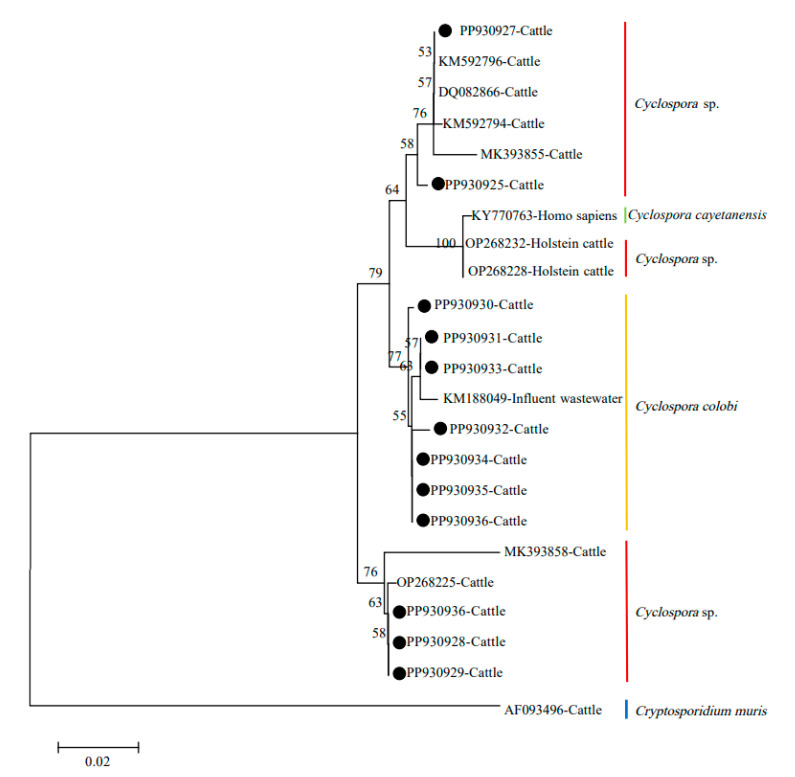
Analysis of phylogenetic relationship of *Cyclospora* spp. in cattle in Shanxi Province using neighbor-joining (NJ) in MEGA 7.0 [15]. Sequences obtained in the present study are marked with a black circle.

In the present study, 12 distinct sequences were identified through Sanger sequencing analysis. The sequences generated during the current study are available in the GenBank database (PP930925–PP930936). Seven sequences (PP930930–PP930936) showed a high level of sequence identity (greater than 98.8%) with the *C. colobi* sequence (KM188049) in the GenBank database, and the remaining sequences were more similar to that of the *Cyclospora* spp. isolated from the cattle. In a neighbor-joining analysis, five *Cyclospora* spp. isolated from cattle in the present study grouped with other cattle *Cyclospora* spp. reported previously, whereas the remaining seven samples grouped with *C. colobi* (Figure 2). All of the *Cyclospora* spp. identified in the present study were relatively distant from the human pathogen *C. cayetanensis*.

## 4. Discussion

The methods for the examination of *Cyclospora* spp. include morphological and molecular detection techniques [2]. Compared with microscopic examination, the application of molecular methods for the diagnosis of *Cyclospora* spp. infection has various strengths, such as high sensitivity. A previous study showed that *C. cayetanensis* was not detected in any of the fecal samples of 291 patients by traditional microscopy, whereas 5 fecal samples were detected to be positive by PCR [21]. In addition, a molecular method such as PCR can allow for species-level identification [2,22]. In 1996, a nested PCR targeting the SSU rRNA gene segment of *Cyclospora* spp. was developed [23]. Subsequently, an RFLP analysis of PCR products was developed based on the nucleotide differences in the amplified region to distinguish *Cyclospora* spp. from *Eimeria* spp. [24]. To date, the restriction enzymes used in the RFLP analysis included *Mnl*I, *Kpn*I, and *Kpn*2I [2,15,19]. After careful consideration of the digestion fragment sizes, clarity, and readily differentiation of all closely related species, we chose *Kpn*2I (synonym *Bsp*EI) to digest the PCR products of the SSU rDNA amplified from the cattle fecal samples in this study. The results obtained in the present study confirmed the presence of *Cyclospora* spp. in cattle in Shanxi Province, North China, and the overall prevalence was 2.1%.

So far, limited data are available regarding the prevalence of *Cyclospora* spp. in ruminants. The prevalence of *Cyclospora* spp. in dairy cattle in Shanxi Province was 1.8%, which was slightly lower than that reported in Holstein cattle (2.5%) in Yunnan Province, China [15]. The overall prevalence of *Cyclospora* spp. in cattle (2.1%) in Shanxi Province was slightly higher than that found in goats (1.9%), but lower than that found in sheep (3.1%) in Tamil Nadu, India [25]. Region, age, sex, and breed were not identified to be risk factors associated with the *Cyclospora* prevalence in cattle in the present study, which is consistent with the results of a previous study [15]. Of note, there was no statistically significant difference in the prevalence of *Cyclospora* spp. infection in cattle between the two age groups (*p* = 0.053); however, a higher prevalence was observed in animals older than 12 months, which may indicate that older animals are more likely to be exposed to the parasites.

Two sequences obtained in this study were identical to those of *Cyclospora* isolates deposited previously in GenBank, which may be indicative of strong clonality, regardless of the geographical location. In addition, sequence variation was observed in the SSU rRNA gene of *Cyclospora* isolates obtained in this study, which was also reported in a previous study [15]. Sequencing and phylogenetic analysis showed that seven *Cyclospora* isolates represented *C. colobi*, which has been reported in nonhuman primates [20,26].

The results of a previous study suggested that domestic animals are not reservoir hosts for *C. cayetanensis*, because no oocysts morphologically compatible with *C. cayetanensis* were detected in fecal samples from 327 domestic animals (such as pigs, cattle, horses, and goats) [12]. Also, the human species *C. cayetanensis* was not found in cattle in the present study based on SSU rRNA sequences. The presence of *C. cayetanensis* in fecal samples from cattle and dogs has been reported [14,15]. However, animals shedding oocysts in their feces does not necessarily indicate that they are reservoir hosts, because the oocysts ingested by animals may pass through their digestive tract without causing infection. Further investigations based on a biopsy from the small intestine are needed to confirm whether cattle serve as paratenic hosts or reservoirs for *C. cayetanensis*.

*Eimeria* infections are common in cattle worldwide [27]. In mainland China, the prevalence of *Eimeria* in cattle ranged from 4.6% to 87.5% [28]. In our present study, the nested PCR results showed the presence of a specific band of approximately 500 bp in size in 473 cattle fecal samples. Of the 473 positive samples, RFLP analyses revealed that only 16 represented *Cyclospora* spp.; 1 positive fecal sample suggested co-infection of *Eimeria* spp. and *Cyclospora* spp., consistent with the results of a previous study [15]; and the rest represented *Eimeria* spp., indicating a high prevalence of *Eimeria* spp. in cattle in Shanxi Province.

## 5. Conclusions

The present study revealed an overall 2.1% prevalence of *Cyclospora* spp. in cattle in Shanxi Province for the first time, and region, age, sex, and breed were not found to be significantly associated with *Cyclospora* infection. Twelve representative sequences were obtained, with sequence identity ranging from 94.3 to 98.1%. Among them, seven sequences were identified as *C. colobi* and five sequences represented other *Cyclospora* spp. These findings have important implications for carrying out intervention measures against *Cyclospora* spp. infection in cattle and other animals.

## Figures and Tables

**Table 1:** Factors associated with prevalence of *Cyclospora* spp. in cattle in Shanxi Province, North China.

Factor	Category	No. Examined	No. Positive	Prevalence% (95% CI)	OR (95%)	*p*-Value
Region	Qi County	288	5	1.7 (0.2–3.2)	1.7 (0.3–9.1)	0.196
	Shanyin County	200	2	1.0 (−0.4–2.4)	1	
	Jishan County	273	9	3.3 (1.2–5.4)	3.4 (0.7–15.8)	
Age	Month < 12	269	2	0.7 (−0.3–1.8)	1	0.053
	Month ≥ 12	492	14	2.8 (1.4–4.3)	3.9 (0.9–17.3)	
Sex	Male	242	5	2.1 (0.3–3.9)	1	0.962
	Female	519	11	2.1 (0.9–3.4)	1.0 (0.4–3.0)	
Breed	Dairy cattle	385	7	1.8 (0.5–3.1)	1	0.580
	Beef cattle	376	9	2.4 (0.8–3.9)	1.3 (0.5–3.6)	
Total		761	16	2.1 (1.1–3.1)		

## Data Availability

The datasets supporting the results of this article have been submitted to GenBank, and the accession number is shown in the article.
